# The Impact of Case Diagnosis Coverage and Diagnosis Delays on the Effectiveness of Antiviral Strategies in Mitigating Pandemic Influenza A/H1N1 2009

**DOI:** 10.1371/journal.pone.0013797

**Published:** 2010-11-03

**Authors:** Joel K. Kelso, Nilimesh Halder, George J. Milne

**Affiliations:** School of Computer Science and Software Engineering, University of Western Australia, Crawley, Australia; Singapore Immunology Network, Singapore

## Abstract

**Background:**

Neuraminidase inhibitors were used to reduce the transmission of pandemic influenza A/H1N1 2009 at the early stages of the 2009/2010 pandemic. Policies for diagnosis of influenza for the purposes of antiviral intervention differed markedly between and within countries, leading to differences in the timing and scale of antiviral usage.

**Methodology/Principal Findings:**

The impact of the percentage of symptomatic infected individuals who were diagnosed, and of delays to diagnosis, for three antiviral intervention strategies (each with and without school closure) were determined using a simulation model of an Australian community. Epidemic characteristics were based on actual data from the A/H1N1 2009 pandemic including reproduction number, serial interval and age-specific infection rate profile. In the absence of intervention an illness attack rate (AR) of 24.5% was determined from an estimated R_0_ of 1.5; this was reduced to 21%, 16.5% or 13% by treatment-only, treatment plus household prophylaxis, or treatment plus household plus extended prophylaxis antiviral interventions respectively, assuming that diagnosis occurred 24 hours after symptoms arose and that 50% of symptomatic cases were diagnosed. If diagnosis occurred without delay, ARs decreased to 17%, 12.2% or 8.8% respectively. If 90% of symptomatic cases were diagnosed (with a 24 hour delay), ARs decreased to 17.8%, 11.1% and 7.6%, respectively.

**Conclusion:**

The ability to rapidly diagnose symptomatic cases and to diagnose a high proportion of cases was shown to improve the effectiveness of all three antiviral strategies. For epidemics with R_0_< = 1.5 our results suggest that when the case diagnosis coverage exceeds ∼70% the size of the antiviral stockpile required to implement the extended prophylactic strategy decreases. The addition of at least four weeks of school closure was found to further reduce cumulative and peak attack rates and the size of the required antiviral stockpile.

## Introduction

Treatment and prophylaxis with antiviral drugs is a core strategy in the influenza pandemic preparedness plans of many countries [1], [2], [3] and was utilized for the first time during the 2009 A/H1N1 pandemic [4].

The efficacy of antiviral drugs for treatment and prophylaxis has been demonstrated in trials, as analysed in [5]. In addition to reducing the severity and duration of symptoms [6], neuraminidase inhibitors also reduce both infectiousness of treated individuals and susceptibility of exposed individuals undergoing prophylaxis [5], preventing secondary transmission and thus potentially reducing the impact of the epidemic. Modelling studies oriented to H5N1 have been used to determine their effectiveness in reducing illness attack rates; examples include reducing illness amongst health care workers [7], [8] and in the wider community [9], [10], [11], [12], [13].

We have expanded on previous modelling studies by simulating, in detail, the effect of several key aspects of antiviral interventions. These include the effect of *delaying diagnosis* and the *ratio of diagnosed to undiagnosed symptomatic cases*, as well as the subsequent antiviral treatment and (possible) prophylaxis. We simulated epidemics in a community of 30,000 individuals, basing the characteristics of the influenza strain on those of the 2009 pandemic as estimated from actual pandemic data including reproduction number [14], [15], [16], [17], [18], serial interval [14], [15], and age-specific attack rate profile [19].

The 2009 A/H1N1 pandemic revealed that strategies for using antiviral drugs differed markedly between and within countries [4]. One of these differences was whether antiviral drugs were used solely for treatment or also for prophylaxis. Prophylaxis strategies also differed in terms of the extent of the contact group at which the prophylaxis was targeted; that is, whether it was household members only or whether it was extended to include workplace or school contacts. The decision to use antivirals in a prophylactic capacity (and if so, how extensively) will clearly determine both the population-level effect of the intervention and the magnitude of the antiviral resources needed. We simulated three increasing scales of antiviral usage: treatment only, treatment plus household prophylaxis, and treatment plus household prophylaxis plus prophylaxis of workplace or school class contacts.

Another difference in the application of antiviral drugs involved the methods used to determine who should receive treatment, and as a consequence who should also receive prophylaxis. Some countries (e.g. some states in Australia) initially required laboratory testing before initiation of a treatment regime (and the possible prophylaxis of a contact group), while others only required diagnosis of an influenza-like illness (ILI) by a medical practitioner. In other countries (e.g. the United Kingdom) diagnosis could be conducted over the telephone by a health-care worker with immediate authority for the prescription of antiviral drugs. Assuming that infected individuals seek medical attention upon the development of symptoms, the diagnosis procedure adopted is a key determiner of the time delay between symptom onset and initiation of treatment (and possible prophylaxis). Since viral shedding in an infected person occurs around the time of peak symptoms [20], diagnosis delay will strongly influence the effectiveness of antivirals in interruption of transmission. We simulated diagnosis delays ranging from immediate (less than 6 hours), which might be possible in the case of the telephone system described above, to 48 hours after symptoms appearing, which might be a plausible (though unlikely) turn-around time for a heavily loaded testing laboratory.

In addition to variations in these operational aspects of antiviral delivery, a key observation of the 2009 pandemic was the difficultly in ascertaining all the cases, and therefore the proportion of infected cases to which antiviral interventions were being applied (which we refer to as the diagnosis coverage). We simulated such diagnosis coverages ranging form 10% through to 100%.

As school closures were a common adjunct to antiviral intervention policies, we also simulated all antiviral interventions with and without concurrent school closures.

In the case of a newly emerged and highly virulent influenza strain, a key aim of public health policy will be to contain infection spread – that is, reduce the rate of new infections to a very low level – either to prevent an epidemic or to buy time for a vaccine to be developed and distributed. Current pandemic planning calls for antiviral drugs to be used as part of such a containment response, so understanding the effects of operational issues pertaining to planned antiviral interventions is vital. Our simulation experiments allowed us to quantify reductions in the overall illness attack rate and in the maximum daily case load under a range of diagnosis delays and diagnosis coverages for both treatment-only and treatment plus prophylaxis strategies. Detailed examination of these factors also permits us to establish how these two diagnosis criteria impact on the required size of an antiviral stockpile.

## Methods

### Simulation Model

We used an individual-based model of a real community in Western Australia (Albany) with a population of approximately 30,000 to simulate the dynamics of the 2009 influenza pandemic. We used census, state and local government data to construct a human contact network involving households, schools, childcare centres, workplaces and a regional hospital. The simulation period was divided into 12 hour day/night cycles; during each cycle the nominal location of every person was determined, and individuals occupying the same location were assumed to come into potential infective contact. In addition, community interaction was modelled by assuming that active individuals would contact other active individuals each day, with contact being random but biased towards contact between people with nearby home locations.

This model was previously developed to determine the effectiveness of social distancing and vaccination measures for a possible future H5N1 pandemic [21], [22], [23], and was subsequently used to examine antiviral and school closure interventions that were employed in the A/H1N1 2009 pandemic [13]. We have further refined this model to include the ability to simulate diagnosis delays and coverages, and to reflect the biology of the A/H1N1 2009 influenza strain according to information available in early 2010.

Transmission of infection between infectious and susceptible individuals who came into infective contact was resolved stochastically. The probability of transmission was calculated as a function of the state of the infectious (I_i_) and susceptible (I_s_) individuals involved at the time of contact, as given by:



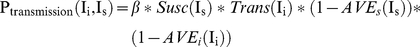
Each factor contributing to the transmission probability is described below. The basic transmission probability (β), capturing the infectivity of the virus strain, was chosen to give an unmitigated epidemic with a reproduction number R_0_ of 1.5. We also determined alternative basic transmission probabilities that gave epidemics with R_0_ values of 1.2, 2.0 and 2.5 – the rationale for selecting these values is presented in the Discussion section.

To achieve a realistic age specific infection rate, age-specific susceptibility parameters (the function *Susc* appearing above) were calibrated to achieve an age-specific attack rate similar to that of the A/H1N1 2009 pandemic. This was achieved using the following procedure. Using the age distribution of cases reported to the EDCD [19] (based on European Union influenza surveillance data for the period April to September 2009) and the age demographics of the Albany model (which are similar to those of Europe), we calculated an age-specific attack rate profile. We then determined transmission probability susceptibility parameter values (*Susc*) for each age group in order to give an epidemic that matched this attack rate profile. The basic virus transmission probability parameter was then adjusted (keeping age-specific susceptibilities constant) to produce epidemics with target reproduction numbers, as described above.

**Figure 1:**
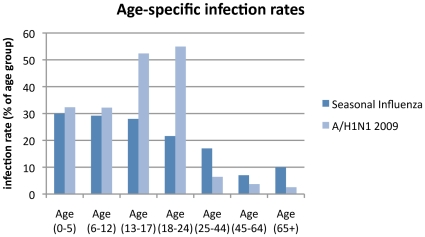
Age-specific infection rate profiles for seasonal and A/H1N1 2009 influenza used to calibrate age-specific susceptibility. The proportion of each age group infected in a baseline (unmitigated) epidemic is shown for seasonal influenza and for A/H1N1 2009. In both cases age demographics are those of the Albany model, and final infection rates are 17% (corresponding to a 13% final illness attack rate).

In order to determine if this assumption has an important influence on the effectiveness of antiviral strategies, we repeated our simulations with an alternative set of age-specific susceptibility parameters that gave rise to age-specific attack rates similar to those of seasonal influenza (parameters were calibrated to serologic infection rates reported for H3N2 in 1977–1978 in Tecumseh, Michigan [24]). The main difference between the two age-specific attack rate profiles is the greater numbers of cases in the 12–24 age group for A/H1N1 2009, but fewer cases in older age groups, compared to seasonal influenza. The age-specific infection rate profiles used to determine age-specific susceptibilities for the two different assumptions are shown in Figure 1.

Infected individuals were fully infectious (i.e. *Trans*(I_i_)  = 1.0) from 36 hours after infection (when symptoms were deemed to appear) to 84 hours after infection; and less (half) as infectious for the rest of the infectivity period (i.e. *Trans*(I_i_)  = 0.5), which began 12 hours after infection and finished 6 days after infection. This timeline of the progression of individual infection (which we refer to as the *infectivity profile*), in conjunction with other the simulation parameters and the structure of mixing groups, results in a mean serial interval of 2.3 days (standard deviation 1.6 days), which was consistent with the A/H1N1 2009 pandemic as estimated in [14], [15]. The serial interval was calculated by determining the time between the event when an individual became infected, and subsequent infection event due to that individual infecting another, averaged over all infected individuals during a simulation run.

The peaked infectivity profile described above is an approximation of the viral shedding distribution documented in [20]. In order to examine the sensitivity of our results to this choice of infectivity profile we conducted an analysis using 4 additional alternative infectivity profiles. For each alternative infectivity profile, a corresponding β (basic transmission probability) value was determined so that the resulting no-intervention epidemics all had an R_0_ value of 1.5, and the simulation experiment series was repeated. The full details of the alternative infectivity profiles are given in Supporting Information Text S1. Although there is evidence that infectiousness (as well a susceptibility) is age-specific, we have not included this effect in our model.

We assumed that 32% of infected adults (20% of children) would experience asymptomatic infection [25], and that 50% of symptomatic adults (90% of children) would isolate themselves in their household for the duration of their infection. In the baseline epidemic, this leads overall to 23% of infections being asymptomatic, which is consistent with an estimate for the 2009 A/H1N1 pandemic of 22% [26]. We assumed that an average of one new infection per day was stochastically introduced into the population during the whole period of the simulations.

As with earlier work other parameter values such as community contact rates and school class sizes were selected to give plausible values for in-household versus out-of-household transmission [21].

### Antiviral Efficacy

We assumed that the probability of infection transmission during an infectious contact was reduced by 66% if the infected individual was undergoing antiviral treatment (i.e. *AVE_i_*(I_i_)  = 0.66) [5], [27], and that treated individuals experienced a 1-day reduction in illness duration [28]. During cycles in which antiviral treatment is *not* in effect, which could be because no antiviral treatment strategy was being simulated, or the individual was not symptomatic, or was not diagnosed, or treatment had not yet begun due to diagnosis delay, AVE_i_(I_i_) was set to 0.

Note that in the case that an individual became infected while undergoing antiviral prophylaxis but did not receive treatment, either because they experienced asymptomatic infection, or because they were not diagnosed (see Diagnosis Delay and Coverage below), the same AVE_i_ reduction in infectiousness was applied during the prophylaxis period.

Similarly, the transmission probability was reduced by 85% if the susceptible individual was undergoing antiviral prophylaxis (i.e. *AVE_s_*(I_s_)  = 0.85) [6], and were 50% less likely to experience symptomatic illness if they did become infected [28]. For individuals not undergoing treatment or prophylaxis, the respective *AVE_i_* and *AVE_s_* parameters were set to 1.0.

We further examined the possibility that the efficacy of reducing infectivity is dramatically reduced if treatment is delayed by conducting a sensitivity analysis with the alternate assumption that AVE_i_ declined exponentially with the length of time between symptoms developing and AV administration, with AVE_i_ reduced by one half for each 24 hour delay. Figures illustrating the action of AVE_i_ for various diagnosis delays are contained in Supporting Information Text S1

### Antiviral Strategies

We analysed three different antiviral intervention strategies that were used (variously) in Australia, the United Kingdom and the USA during the early stages of the 2009 influenza pandemic. These strategies were:

1. Treatment-only (T): Diagnosed individuals received antiviral drug treatment.
2. Household prophylaxis (T+H): Diagnosed individuals received antiviral treatment and all household members were given antiviral drugs for prophylaxis.
3. Extended prophylaxis (T+H+E): Here the prophylactic use of antiviral drugs was extended to a wider group of contacts, with prophylaxis given to class members (if the diagnosed person is school pupil or teacher) or to workplace contacts (if the case was diagnosed in a workplace location), in addition to their household members.

Antiviral treatment involved diagnosed individuals receiving two doses taken daily for 5 days; antiviral prophylaxis consisted of one dose taken daily for 10 days.

Note that by “diagnosis” we do not necessarily mean laboratory confirmed diagnosis; merely that an individual sought medical attention and a decision to administer antivirals was made. Note that for the prophylaxis scenarios, we assumed that an individual who became infected (and was diagnosed) during prophylaxis would switch to a new full-length antiviral treatment course. We also assumed that a person would receive at most two prophylactic courses; and that they would not receive prophylaxis if they had previously experienced symptomatic infection.

### School Closure

For each of these strategies, we simulated epidemics with and without school closure (SC). We assumed that closure of each school was triggered following diagnosis of two cases in the school, whereupon the school was closed for two weeks, with each school closing on at most two occasions for a maximum total of 4 weeks. School closures were applied to primary and secondary schools but not to childcare facilities or adult education institutions. We assumed that teachers and pupils affected by school closure would not attend their regular school hub during the daytime cycle but instead dwelt at home, coming into contact with other individuals present in the household. Individuals so affected were assumed to make their usual community contacts at during the day, but made no additional (compensatory) contacts.

### Diagnosis Delay and Coverage

We simulated *diagnosis coverages*, that is, the percentage of those who experience symptomatic infection who are actually diagnosed, ranging from 10% through to 100% in 10% increments.

We define the *diagnosis delay* to be the period from when an individual first experiences symptoms to the time when they receives antivirals. We assumed that antiviral treatment or prophylaxis began at the time diagnosis was made. We simulated diagnosis delays ranging from immediate (less than 6 hours after the appearance of symptoms) up to 48 hours, in 12 hour increments.

We assumed that the actual time of diagnosis relative to the time of infection or symptom onset may be caused by a variety of factors; delay in individuals seeking medical attention, access to health care facilities, delay in laboratory diagnosis, or availability of antiviral drugs.

## Results

In the absence of interventions our simulated baseline epidemic had an R_0_ of 1.5, a final attack rate (AR) of 24.5%, and a serial interval of 2.32 days. Additional epidemic characteristics, and characteristics for alternate baseline epidemics with R_0_ values of 1.2, 2.0, and 2.5 can be found in Supporting Information Table S1. The rationale for selecting these values is presented in the Discussion section. Results for all simulated epidemics were determined from the average of 40 individual simulation runs, each with stochastic choices made using a different random number sequence.

**Figure 2:**
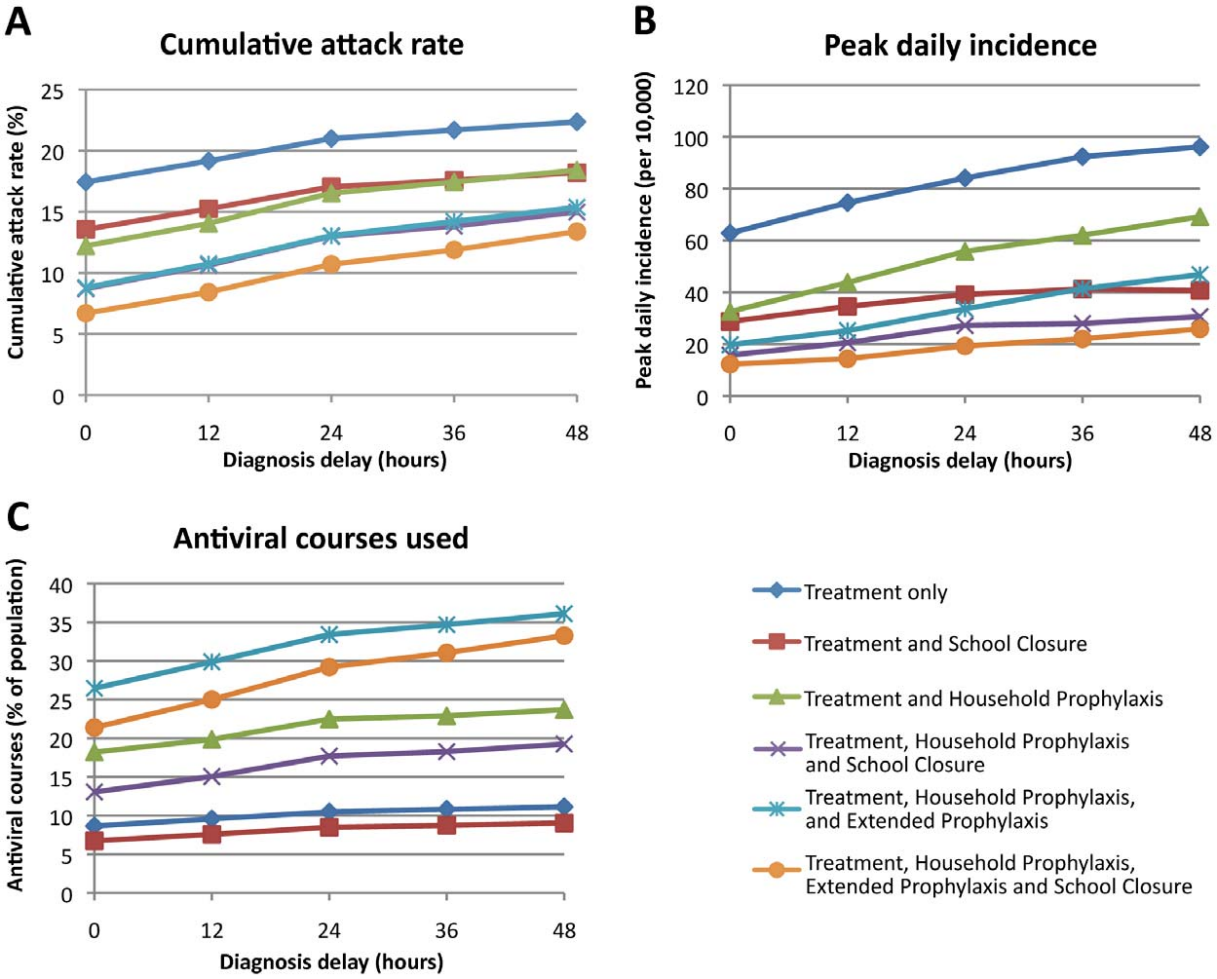
Outcome of six antiviral intervention strategies as a function of diagnosis delay. Three outcomes are reported: (A) cumulative illness attack rate, (B) peak daily incidence (per 10,000 population), and (C) number of antiviral courses used as a percentage of the population size. We assumed that antiviral treatment or prophylaxis began at the time diagnosis was made and that 50% of symptomatic cases would be diagnosed.

**Figure 3:**
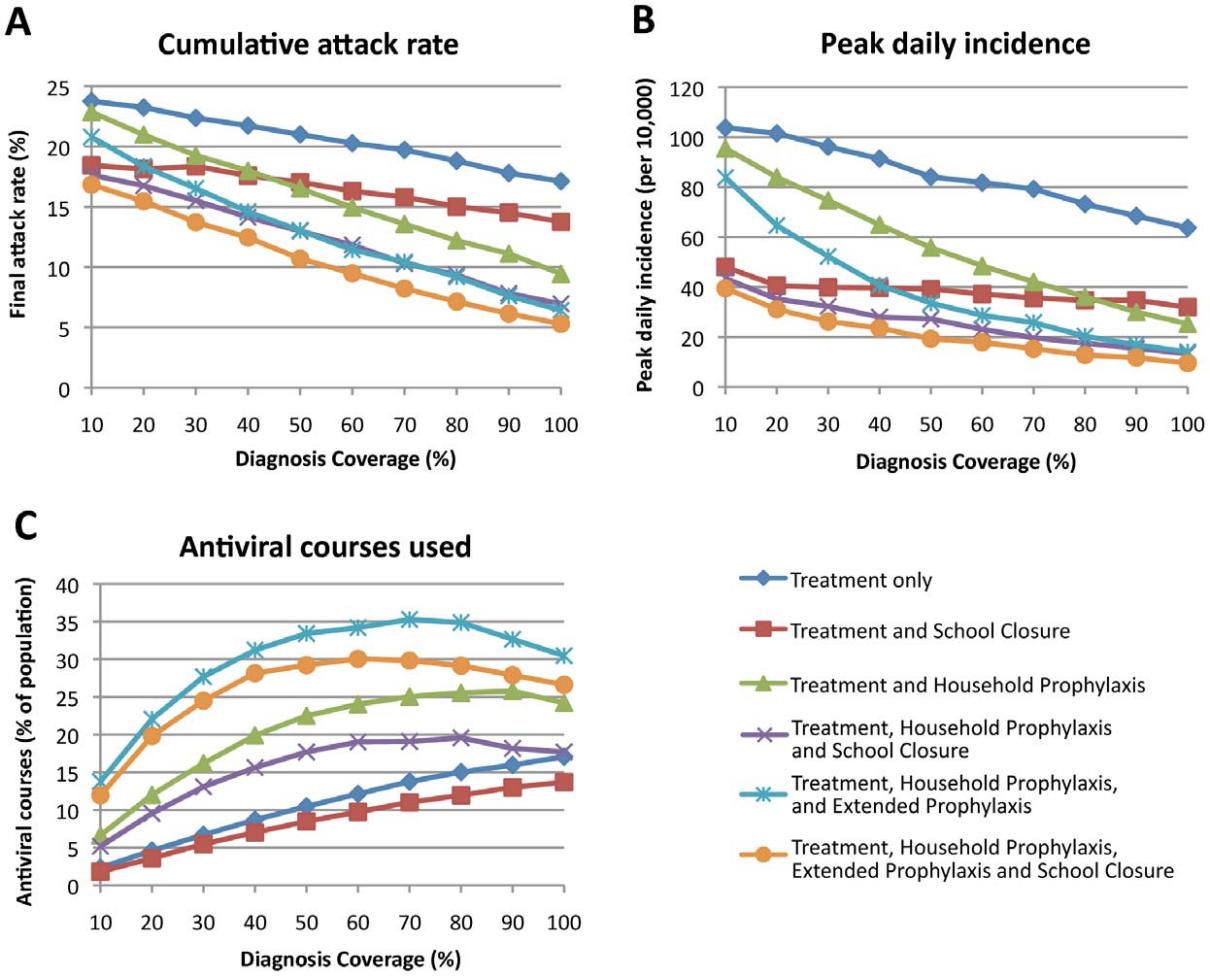
Outcome of six antiviral intervention strategies as a function of diagnosis coverage. Three outcomes are reported: (A) cumulative illness attack rate, (B) peak daily incidence (per 10,000 population), and (C) number of antiviral courses used as a percentage of the population size. We simulated percentages of symptomatic individuals being diagnosed ranging from 10% to 100% in 10% increments. We assumed that the delay between symptoms appearing and antiviral treatment or treatment plus prophylaxis was 24 hours.

Several patterns of results held across all intervention scenarios. Greater prophylactic use of antivirals always resulted in greater reductions in AR: strategy T+H+E was better than T+H which in turn was better than T (see Figure 2A and Figure 3A).

The pattern of peak daily incidence reductions was the same as for final AR reductions, with daily incidence reductions being proportionally larger than final AR reductions (see Figures 2B and 3B).

The addition of 4 weeks school closure to any antiviral strategy consistently gave an additional decrease in antiviral usage, AR, and peak daily incidence.

### Impact of Diagnosis Delay

We simulated a range of diagnosis delays from zero to 48 hours, assuming a 50% diagnosis coverage. Results for the three antiviral drug strategies, with and without 4 weeks of school closure, are shown in Figure 2. Figure 2A shows that delaying antiviral treatment (and related prophylaxis if used) resulted in an approximately linear increase in AR for all strategies.

For the best antiviral strategy (T+H+E) the AR ranged from 8.8% with prompt diagnosis (no delay between symptom appearance and antiviral administration) to 15.4% with a 48 hour delay; for the T strategy the AR increased from 17.4.0% to 22.4% over the same range of delays.

Assuming zero-delay diagnosis, a further 3.9%, 3.5% or 2.1% reduction in the final attack rate resulted from the addition of 4 weeks of school closure to the T, T+H and T+H+E strategies respectively. The additional reduction in attack rate due to school closure is very similar for diagnosis delays ranging from 0 to 48 hours.

**Figure 4:**
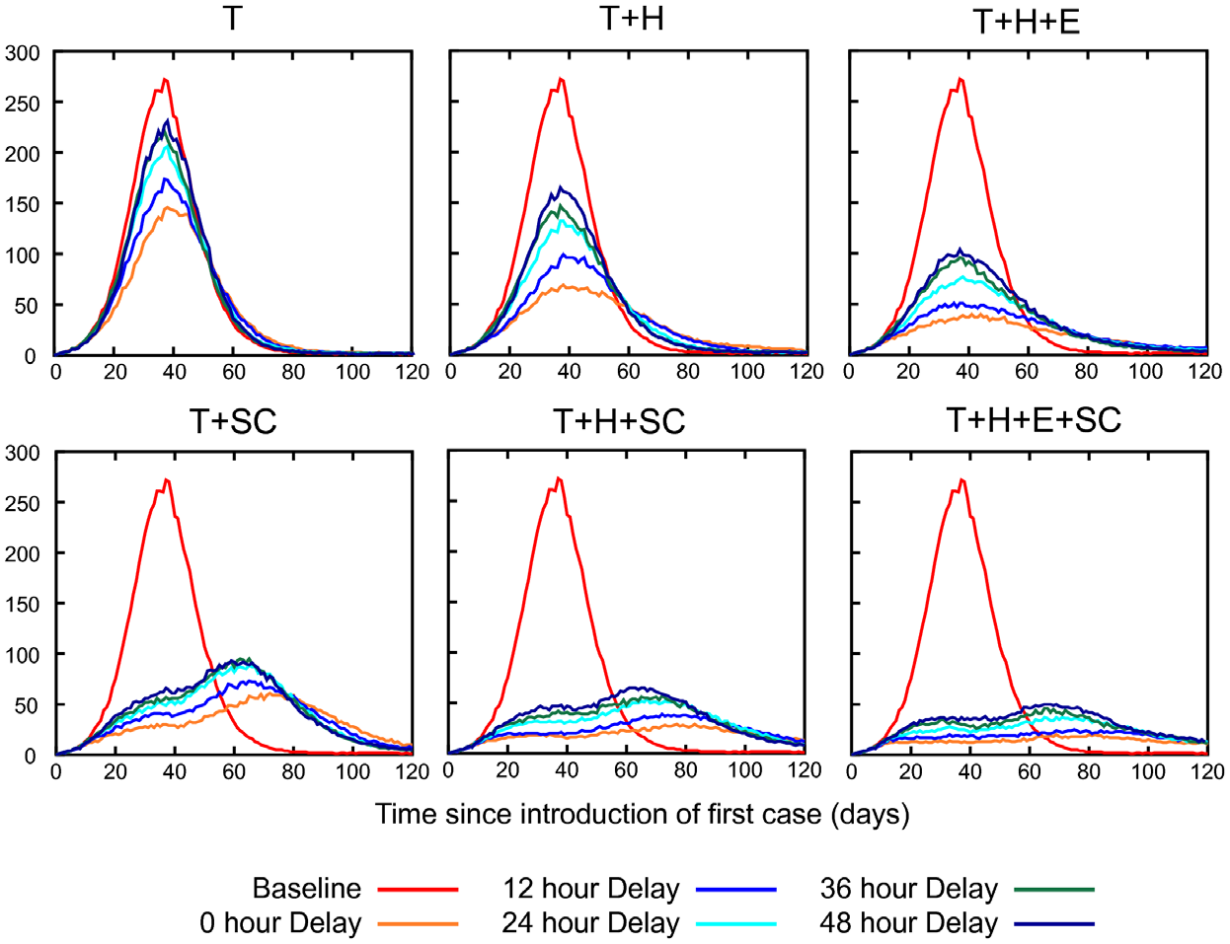
Daily incidence epidemic curves for various delays in antiviral treatment and/or prophylaxis. Interventions are abbreviated as follows: treatment only (T), household prophylaxis (H), extended prophylaxis (E), 4 weeks school closure (SC). We assumed that 50% of symptomatic cases would be diagnosed. Schools were assumed to close upon the diagnosis of 3 cases in the school for a period of two weeks. Each school closed a maximum of 2 times for a total of 4 weeks.

The peak daily incidence is also reduced by prompt usage of antiviral drugs. If there is no delay between symptom appearance and diagnosis the maximum number of symptomatic cases per day is reduced by 46 (109 to 63), 76 (109 to 33) and 89 (109 to 20) per 10,000 population using the T, T+H and T+H+E strategies respectively. The addition of school closure can avoid a further 34, 17 and 8 cases per 10,000 of the population respectively. If antiviral treatment and prophylaxis were started 24 or 48 hours post symptom appearance, all strategies are less effective in reducing the peak daily incidence, with treatment-only being affected most adversely, as shown Figure 2B. Figure 4 shows daily incidence epidemic curves for various intervention strategies and diagnosis delays. The characteristic “double hump” appearing in the school closure epidemic curves is due to schools re-opening after their maximum 4 weeks of school closure, and the subsequent acceleration of the epidemic at that point. This indicates that the school closure component of the modelled interventions could be more effective if optimally timed (this phenomena is considered further in the Discussion section).

### Impact of Diagnosis Coverage

**Figure 5:**
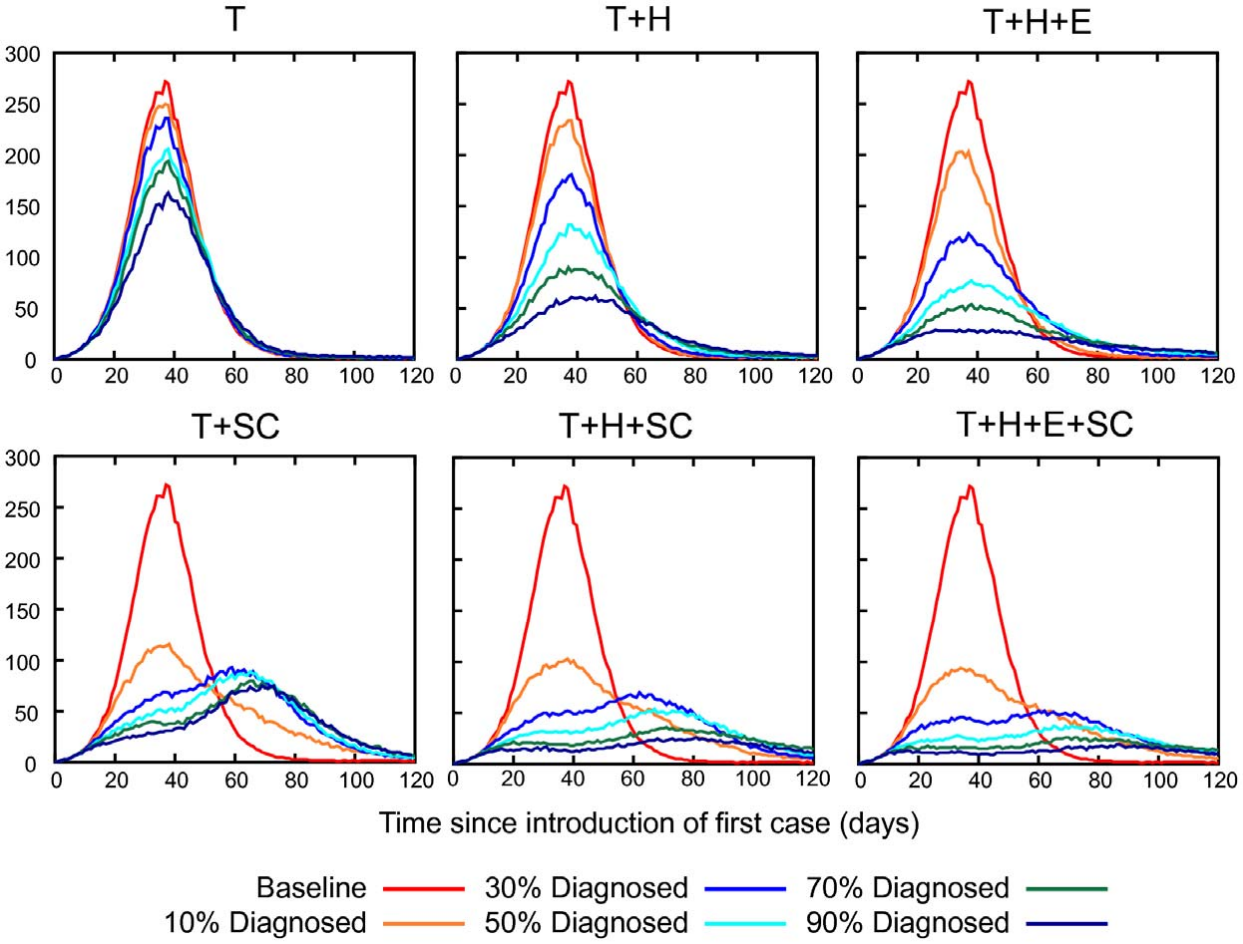
Daily incidence epidemic curves for various diagnosis coverages. Interventions are abbreviated as follows: treatment only (T), household prophylaxis (H), extended prophylaxis (E), 4 weeks school closure (SC). We assumed that the delay between symptoms appearing and antiviral treatment or treatment plus prophylaxis was 24 hours. Schools were assumed to close upon the diagnosis of 3 cases in the school for a period of two weeks. Each school closed a maximum of 2 times for a total of 4 weeks.

A range of diagnosis coverages for each of the three antiviral drug strategies were analysed using a realistic diagnosis delay of 24 hours. As might be expected, our results indicate that a higher case diagnosis coverage will reduce the final illness attack rate and the peak daily incidence, as shown in Figures 3A and 3B. With a minimum diagnosis coverage of 10% none of the antiviral drug strategies can contain the epidemic i.e. reduce the illness attack rate to less than 10% of the population. The final attack rates are 23.8%, 22.9% and 20.8% (compared to the unmitigated attack rate of 24.5%), and the peak daily incidence rates are 104, 95 and 84 per 10,000 (compared to the unmitigated value of 109) following administration of the T, T+H and T+H+E strategies respectively. With a 50% diagnosis coverage the attack rates are 21.0% (down 3.5% from 24.5%), 16.5% (down 8.1%) and 13.0% (down 16.4%) respectively. Adding school closure reduced the attack rate further, to 17.1%, 13.0% and 10.7% respectively, showing the benefit of this layered approach. Figure 5 shows daily incidence epidemic curves for various intervention strategies and diagnosis delays. We summarize the impact of different diagnosis delays and diagnosis coverages on the overall illness attack rate and the peak daily incidence in Table 1.

**Table 1:** Outcome of antiviral epidemic measures with various diagnosis delays and coverages.

	final symptomatic attack rate (%)						peak daily incidence (per 10,000)					
baseline	24.5						109					
intervention strategy	diagnosis delay			diagnosis coverage			diagnosis delay			diagnosis coverage		
	0 h	24 h	48 h	10%	50%	90%	0 h	24 h	48 h	10%	50%	90%
T	17.4	21.0	22.4	23.8	21.0	17.8	63	84	96	104	84	68
T+SC	13.6	17.1	18.2	18.4	17.1	14.5	29	39	41	48	39	35
T+H	12.2	16.5	18.4	22.9	16.5	11.1	33	56	69	95	56	30
T+H+SC	8.7	13.0	15.0	17.6	13.0	7.8	16	27	31	43	27	15
T+H+E	8.8	13.0	15.4	20.8	13.0	7.6	20	34	47	84	34	17
T+H+E+SC	6.7	10.7	13.4	16.8	10.7	6.1	12	19	26	39	19	12

Final symptomatic attack rate (as % of population) and peak daily symptomatic incidence (per 10,000) are given for different intervention strategies, diagnosis delays and diagnosis coverages. Intervention strategies are abbreviated as follows: T – antiviral treatment of diagnosed cases, H – prophylaxis of household of diagnosed cases, E – prophylaxis of school or work contacts of diagnosed cases, SC – four weeks of school closure. Where diagnosis delay differs from 24 hours, diagnosis coverage is 50%; where diagnosis coverage differs from 50%, diagnosis delay is 24 hours.

### Impact on Required Number of Antiviral Courses

For all intervention scenarios we found that wider prophylactic use of antivirals resulted in greater numbers of antiviral courses used, with the T+H+E strategy using the most and T using the least. We also found that the addition of school closure to any strategy always resulted in the use of fewer courses.

Figure 2C shows that longer diagnosis delays resulted in greater antiviral usage, with the effect being largest for the prophylaxis strategies. For the T+H+E+SC strategy, the required number of antiviral courses ranged from 21.4% with zero delay to 33.3% with a 48 hour delay; for the T+SC strategy the number of courses required varied from 6.7% to 9.0% over the same range of delay.

As shown in Figure 3C, the effect of diagnosis coverage on the required number of antiviral courses differed qualitatively between the treatment-only and prophylaxis strategies. For T+SC the number of doses required increases linearly with diagnosis coverage, from 1.8% with a diagnosis coverage of 10% to 13.7% with a diagnosis coverage of 100%. For T+H+E+SC the number of courses required increases rapidly to a peak of 30.1% at a diagnosis coverage of 60%; beyond this diagnosis coverage threshold the number of courses required falls, reaching 26.6% at a diagnosis coverage of 100%. For T+H+SC the number of required courses increases more slowly than T+H+E+SC but more rapidly than T+SC, up to a plateau at a diagnosis coverage of 80%, with a fall from 19.6% at a diagnosis coverage of 80% to 17.7% at a diagnosis coverage of 100%.

### Sensitivity to Reproduction Number

Our results reported above assume an influenza epidemic with a basic reproduction number of 1.5. In order to determine the sensitivity of these results to alternate R_0_ assumptions, we repeated our analysis of the effect of diagnosis delays and coverages for epidemics with R_0_ values of 1.2, 2.0 and 2.5. Results of these simulations can be found in Supporting Information Figures S1 and Figure S2; we describe the most significant outcomes below.

As might be expected, higher R_0_ values resulted in higher attack rates and less effective antiviral interventions in proportion to attack rate. Table 2 shows the outcome of each antiviral intervention strategy (with and without school closure) for a range of R_0_ values. These results assume a diagnosis delay of 24 hours and a diagnosis coverage of 50%. For all strategies, increasing R_0_ causes higher final attack rates, higher peak daily incidence and increased antiviral use.

**Table 2:** Outcome of antiviral epidemic measures for epidemics with various reproduction numbers

	R_0_			
intervention strategy	1.2	1.5	2.0	2.5
**cumulative attack rate (%)**				
baseline	12.8	24.5	36.4	43.8
T	8.9	21.0	33.6	41.5
T + SC	5.9	17.1	27.9	36.3
T + H	5.8	16.5	28.6	36.0
T + H + SC	4.1	13.0	23.9	31.3
T + H + E	4.6	13.0	24.4	31.6
T + H + E + SC	3.5	10.7	22.6	29.8
**peak daily incidence (per 10,000)**				
baseline	33	109	274	450
T	20	84	248	430
T + SC	11	39	107	235
T + H	13	56	195	367
T + H + SC	8	27	81	191
T + H + E	9	34	123	259
T + H + E + SC	7	19	62	140
**antiviral usage (number of courses used as % of population)**				
baseline	0.0	0.0	0.0	0.0
T	4.4	10.5	16.8	20.7
T + SC	2.9	8.5	13.9	18.1
T + H	8.5	22.5	34.5	39.7
T + H + SC	6.0	17.7	28.7	34.3
T + H + E	15.3	33.4	47.1	52.7
T + H + E + SC	11.9	29.2	46.4	52.9

Cumulative attack rates (as % of population), peak daily incidences (per 10,000) and number of antiviral courses used (as a % of population size) are given for different intervention strategies and for baseline (i.e. unmitigated) epidemics with four different reproduction numbers (R_0_). Intervention strategies are abbreviated as follows: T – antiviral treatment of diagnosed cases, H – prophylaxis of household of diagnosed cases, E – prophylaxis of school or work contacts of diagnosed cases, SC – four weeks of school closure. In all cases diagnosis coverage is 50% and diagnosis delay is 24 hours.

We found that in terms of diagnosis delay, epidemics of all reproduction numbers followed the same pattern: lowest attack rates and antiviral usage occurred with zero delay, and attack rates and antiviral usage increased essentially linearly with diagnosis delay, up to 48 hours delay. The actual sensitivity to diagnosis delays (that is, the degree to which antiviral effectiveness degraded with increasing delay) depended upon the strategy and R_0_. The degradation in final attacks is shown in Table 3; values ranged from 0.49–1.67 percent per 12 hours delay. We found that for R_0_> = 2.0, the antiviral usage became insensitive to the diagnosis delay.

**Table 3:** Degradation in antiviral effectiveness due to diagnosis delay for epidemics with various reproduction numbers.

	R_0_							
	1.2		1.5		2.0		2.5	
intervention strategy	zero-delay attack rate (%)	attack rate increase (% per 12 hours delay)	zero-delay attack rate (%)	attack rate increase (% per 12 hours delay)	zero-delay attack rate (%)	attack rate increase (% per 12 hours delay)	zero-delay attack rate (%)	attack rate increase (% per 12 hours delay)
baseline	12.8		24.5		36.4		43.8	
T	6.2	1.06	17.4	1.23	31.0	0.93	39.2	0.78
T+SC	4.0	0.81	13.6	1.16	25.5	0.83	33.3	1.04
T+H	3.6	0.97	12.2	1.55	25.0	1.39	32.4	1.46
T+H+SC	2.8	0.54	8.7	1.57	21.5	1.04	28.2	1.34
T+H+E	2.8	0.73	8.8	1.65	20.9	1.33	28.7	1.18
T+H+E+SC	2.4	0.49	6.7	1.67	19.1	1.27	27.1	1.02

Cumulative attack rates (% of population) are given for zero diagnosis delay (i.e. administration of antivirals at the time of symptom appearance) along with the approximate increase in final attack rate that results from each addition 12 hour delay (up to 48 hours). Results are given for different intervention strategies and for baseline (i.e. unmitigated) epidemics with four different reproduction numbers (R_0_). Intervention strategies are abbreviated as follows: T – antiviral treatment of diagnosed cases, H – prophylaxis of household of diagnosed cases, E – prophylaxis of school or work contacts of diagnosed cases, SC – four weeks of school closure. In all cases diagnosis coverage is 50%.

We also found that for the alternate R_0_ scenarios the final attack rates and peak daily incidence varied according to diagnosis coverages in a pattern similar to the R_0_ = 1.5 results.

Since both R_0_ and diagnosis coverages are difficult to estimate with a high degree of certainty, one important statistic is an upper bound on the number of antiviral courses needed to implement a particular intervention strategy. Table 4 gives, for each R_0_ value simulated, the maximum antiviral stockpile needed over all diagnosis coverages, and also the diagnosis coverage for which the maximum occurs. It can be seen that for the treatment-only intervention, or for R_0_ > = 2.0, maximum antiviral usage occurs for a diagnosis coverage of 100% (i.e. increasing diagnosis coverage always require more antivirals); but for prophylaxis strategies at lower R_0_ values maximum antiviral usage plateaus at an intermediate value.

**Table 4:** Maximum antiviral usage for epidemics with various reproduction numbers

	R_0_							
	1.2		1.5		2.0		2.5	
Intervention strategy	max AV usage (%)	diagnosis coverage (%)	max AV usage (%)	diagnosis coverage (%)	max AV usage (%)	diagnosis coverage (%)	max AV usage (%)	diagnosis coverage (%)
T	5.6	90	17.1	100	30.7	100	38.8	100
T+SC	4.2	100	13.7	100	25.1	100	32.8	100
T+H	8.7	70	25.8	90	50.3	100	61.6	100
T+H+SC	6.2	90	19.6	80	42.4	100	51.3	100
T+H+E	15.3	50	35.3	70	60.0	100	71.6	100
T+H+E+SC	12.6	80	30.1	60	58.2	100	72.8	100

Maximum number of antiviral course used (as a % of population size) is given along with the diagnosis coverage that gave rise to that maximum. Results are given for different intervention strategies and for baseline (i.e. unmitigated) epidemics with four different reproduction numbers (R_0_). Intervention strategies are abbreviated as follows: T – antiviral treatment of diagnosed cases, H – prophylaxis of household of diagnosed cases, E – prophylaxis of school or work contacts of diagnosed cases, SC – four weeks of school closure. In all cases diagnosis delay was 24 hours.

### Sensitivity to Age-Specific Attack Rate

As described in the Methods section above, we assumed that the individual age-specific susceptibility to infection was related to the age-specific attack rate (ASAR) of a baseline (unmitigated) epidemic as observed with the 2009 pandemic. In order to determine the effect of this assumption on the effectiveness of antiviral strategies, we repeated our simulations with an alternative set of parameters that gave rise to ASARs similar to those of seasonal influenza [24].

Antiviral diagnosis delay and coverage results based on the seasonal influenza age-specific attack rate assumption can be found in Supporting Information Table S2. Quantitatively, we found that the seasonal ASAR gave higher attack rates than the A/H1N1 2009 ASAR. For example, with R_0_ = 1.5 the baseline (no intervention) final attack rate was 32.5% and the peak daily incidence was 121 per 10,000 compared to 24.5% and 109 per 10,000 for the latter. Although the baseline attack rates were higher, antiviral interventions gave higher *proportional* reductions for the seasonal ASAR assumption. In some cases the prophylaxis strategies reduced attack rates to a level lower than for the A/H1N1 2009 ASAR assumption, despite starting from a higher baseline.

Qualitatively, the effects of antiviral interventions and sensitivity to diagnosis delays and coverages were similar between the two ASAR assumptions: increasing diagnosis delays and diagnosis coverages led to the same patterns of increase in final attack rates, peak daily incidence and antiviral usage.

### Sensitivity to Reduced AVE_i_ Due to Delayed Treatment

An assumption that AVE_i_ is dramatically reduced as a consequence of delayed treatment resulted in only a small additional loss of antiviral effectiveness; approximately 1% increase in the final attack rate. For example, the T+H+E strategy gives a final attack rate of 8.8% assuming the ideal case of there being no delay between symptoms and antiviral treatment. Assuming constant AVE_i_ of 66%, 24 and 48 hour delays give final attack rates of 13.0% and 15.4% respectively; if AVE_i_ is assumed to halve in efficacy with every additional 24 hour delay, the corresponding final attack rates are 14.0% and 16.1% respectively. Full results of the diagnosis delay experiments with the declining AVE_i_ assumption are given in Support Information Text S1.

### Sensitivity to Infectivity Curve and Serial Interval

We conducted an analysis to determine the sensitivity of our results to the individual infectivity profile – that is, the degree to which an infected individual is infectious as a function of time since infection – by repeating our experiments with 4 additional alternative infectivity profiles. Here we report on the results for an alternative infectivity profile that differs from that used in the main results in that the period of maximum infectivity is earlier (beginning 24 rather than 36 hours after infection) and briefer (lasting 36 rather than 48 hours), and for which the maximum level of infectivity is higher relative to the level of infectivity assumed for asymptomatic or post peak infection (being 4 times higher rather than twice as high). Full details and simulation results for all alternative infectivity profiles are reported in Supporting Information Text S1.

The epidemic outcomes for the alternative, more peaked (i.e. having higher hurtosis) infectivity profile exhibited three notable features that contrasted with that of the original infectivity profile.

Firstly the peaked infectivity profile resulted in a shorter serial interval of 1.85 days (standard deviation 0.762 days), compared to 2.31 days (standard deviation 2.88 days) for the original infectivity profile.

Secondly, although parameters for both infectivity profiles were calibrated to give unmitigated epidemics with an R_0_ of 1.5, the final attack rate for the peaked infectivity profile was lower (21.3%) than for the original profile (24.5%).

Thirdly, the peaked infectivity profiles result in greater sensitivity to delay in antiviral treatment, particularly over the first 12 hours after symptom appearance. For example, for the T+H antiviral strategy with the peaked infectivity profile, diagnosis delays of 0, 12 or 24 hours resulted in final attack rates of 6.9%, 11.7% or 13.3%, compared to 12.2%, 14.1% or 16.5% for the same delays for the original profile.

## Discussion

Neuraminidase inhibitors were used in the context of an influenza pandemic for the first time in 2009. Prompted by the observation that a variety of different criteria for distributing antivirals were used in different countries and at different times [4], we evaluated the impact on attack rate reductions arising from delays to diagnosis, and hence the initiation of antiviral use for both treatment and prophylaxis. We also evaluated the impact of varying the percentage of infected individuals who were diagnosed, the diagnosis coverage. Use of actual data from the 2009 pandemic allowed us to investigate these operational details of antiviral interventions in the context of simulated epidemics that matched the A/H1N1 2009 pandemic strain in terms of reproduction number, serial interval and age-specific attack rate profile. We also simulated all antiviral interventions with and without concurrent school closure, as the combination of school with antivirals was used in many locations during the 2009 pandemic and would undoubtedly be used in the future upon the occurrence of a more pathogenic influenza pandemic.

Delaying administration of antiviral treatment and prophylaxis is predicted to result in higher AR and require a larger stockpile of antiviral drugs. Evidence shows that viral shedding (presumed to correlate with infectivity) peaks shortly after the peak in symptoms [20]; delay between symptoms appearing and the beginning of antiviral treatment and prophylaxis thus coincides this period of maximum infectivity. Our results show that a slower, more accurate diagnosis procedure, such as PCR testing, that can distinguish pandemic influenza from other influenza-like illnesses (ILI) is not guaranteed to make better use of an antiviral stockpile; this depends on both the rapidity of diagnosis (the effect of which we quantify) and the prevalence of non-pandemic influenza ILI. The influence of antivirals prescribed for false-positive ILI diagnosis is not simple to predict. Such antiviral usage may have a prophylactic effect, protecting against co-infection with pandemic influenza. If the ILI causing false-positive diagnosis is another influenza strain, antiviral usage may influence the dynamics of this non-pandemic influenza, changing the prevalence of non-pandemic ILI.

An additional point is that while treatment (and possibly household prophylaxis) may be possible via a rapid-diagnosis scheme, this is not the case for extended prophylaxis where contact tracing is necessary: even if contact tracing were to be initiated immediately on symptom appearance, there might be a 24- or 48- hour delay in finding and distributing antivirals to school or workplace contacts. However, since our results show that extended prophylaxis is more effective than treatment-only by a considerable margin, adding prophylaxis, even if delayed, should substantially improve the outcome.

The proportion of symptomatic individuals diagnosed strongly impacts the effectiveness of all strategies. We found that increasing the diagnosis coverage resulted in essentially linear corresponding decreases in the final attack rate. Large decreases in *peak daily* incidence were possible by relatively small increases in diagnosis coverage from 10%: halving the no-intervention peak daily incidence (from 109 per 10,000) could be achieved by diagnosis coverages of 50% or 30% from the household and extended prophylaxis strategies respectively. Adding 4 weeks of school closure resulted in a further halving. The scale of antiviral courses required for these scenarios would preclude using the extended strategy with a high diagnosis coverage for all but those countries with very large antiviral stockpiles, but the potential reduction achieved is considerable.

Estimating diagnosis coverage is difficult, requiring both information on the prevalence (obtained for example through serological surveys) and statistics on clinical diagnosis for the same population. Perhaps due to the mild nature of the 2009 pandemic, diagnosis coverage estimates that have been made are of the order of 5%–10% [29], [30], and are thus at the lower end of the range simulated in this study. However, a pandemic perceived to be more deadly might result in a higher diagnosis coverage, motivating our choice of 50% for a baseline intervention value.

The sensitivity of the stockpile size to diagnosis coverage differs qualitatively between the treatment-only and the prophylactic strategies. At R_0_< = 1.5 increasing the diagnosis coverage beyond these thresholds results in fewer antiviral courses being required. This occurs because at these high diagnosis levels, the prophylaxis strategies suppress infection spread to such an extent that the entire scale of the local epidemic is reduced, consequently requiring fewer, overall antiviral courses. An important caveat is that this applies to epidemics with R_0_ = 1.5; for R_0_ > = 2.0, the required size of the antiviral stockpile increases continuously with an increasing diagnosis coverage.

We also show that school closure is an effective adjunct to all antiviral strategies, reducing final attack rates, peak daily case loads and the number of antiviral courses required. Prior studies [13], [21], [22] with this model indicate that extending school closure periods is increasingly effective, so we may surmise that concurrent school closure periods longer than 4 weeks will be more effective when coupled with an antiviral mitigation strategy. We also note our simulated school closures were not *optimally timed* – Figures 4 and 5 show a characteristic “double hump” in the epidemic curve, which is a due to schools re-opening after 4 weeks of closure and the epidemic consequently accelerating at that point. We assumed that schools would close upon 3 cases were diagnosed in the school; whereas the time or triggering condition that gives the largest reduction in attack rate depends on the transmissibility of the epidemic and the duration of school closure [31]. A previous study using the same simulation model examined the sensitivity of the effectiveness of school closure to various alternative modelling assumptions ([21], Supporting Information).

The reproduction number R_0_ of an epidemic can be difficult to estimate at the outset of an epidemic as it can vary from strain to strain, and from population to population for the same strain [32]. In order to determine the sensitivity of our results to variation in R_0_, we repeated our simulation experiments for epidemics with R_0_ values of 1.2, 1.5, 2.0 and 2.5. We chose to report results for R_0_ = 1.5 (giving a final symptomatic attack rate of 24.5%) in the main paper as our baseline value as this value lies within the range first estimated for the 2009 pandemic from Mexico outbreak data (1.4–1.6) [14] and also within the wider range of subsequent estimates which have ranged from 1.2 to 2.1 [15], [16], [17], [18].

We assumed that differential susceptibility to infection among age groups would lead to age-specific attack rates similar to those of the 2009 pandemic, which differed from seasonal influenza (which it was similar to in many other respects) in that the it exhibited a greater numbers of cases in the 12–24 age group but fewer cases in older age groups [19]. In order to determine the sensitivity of our results to the assumption of 2009-like age-specific attack rate, we repeated our simulation experiments assuming that age-specific susceptibilities were similar to seasonal influenza. There is also the possibility, not included in our model, that infectiousness as well as susceptibility is age-dependant, with children being more infectious than adults. If this is the case then school closure is likely to be more effective than our results indicate.

We found that compared to epidemics with age-specific susceptibilities based on seasonal influenza, the baseline simulated 2009 epidemics exhibited a lower attack rate and lower peak daily incidence; however, *proportional* reduction in attack rates achieved by antiviral interventions were also lower. This indicates that the effectiveness of antiviral interventions may be overestimated if modelling is based on seasonal influenza data. We attribute lower attack rate (compared to seasonal influenza with the same R_0_) to the existence of a subpopulation (the 12–24 age groups) that had a much higher individual susceptibility to infection but who mixed disproportionately with themselves, compared to the larger population. The *qualitative* effectiveness of antiviral interventions was very similar however, with the effects of antiviral strategies, diagnosis delays and diagnosis coverage following the same pattern in both cases.

Our model of antiviral effectiveness assumed that antiviral effectiveness in reducing infectivity (AVE_i_) was constant regardless of when antiviral treatment was initiated. This is possibly too simplistic; it might be the case that antiviral effectiveness declines rapidly as infection develops within the infected individual. We examined an alternative assumption that AVE_i_ drops rapidly with treatment delay, halving with each additional 24 hour delay. We found that under this assumption the *additional* decline in effectiveness of antiviral treatment strategies due to treatment delay was small. It appears that most of the reduction in effectiveness due to treatment delay is simply due to the fact that a proportion of an infected individual's infective duration goes untreated, and that this period, just after symptom appearance, is when many transmission events are concentrated, due to this being a period of high infectivity. Assuming that delayed treatment also resulted in lower AVE_i_ once treatment was initiated did result in a higher attack rate compared to the assumption of constant AVE_i_, but this effect was small compared to the effect described above. Given that antiviral delayed usage results in a smaller effect per course of drugs, an alternative strategy to make use of limited antiviral resources might be to limit antiviral usage to individuals who are either suffering serious complications or seek medical attention immediately on developing symptoms. The current study cannot quantitatively assess this strategy; and since this strategy would require rapid diagnosis the effect of false-positive diagnoses would need to be taken into account.

Another factor that may strongly influence the effectiveness of antiviral strategies is how the infectiousness of an individual changes over time. Our sensitivity analysis demonstrated that if the infectivity profile is sharply peaked around the time of symptom development (and drops off rapidly afterwards), delays in the administration of antivirals are even more detrimental.

Other influenza simulation studies have used a similarly peaked infectivity profile [9], [33]. This profile matches the viral shedding profile data reported in the literature [20], [34], if it is assumed that infectivity is linearly proportional to the measured viral titre. The peaked infectivity profile assumption results in a short serial interval of 1.85 days, compared to 2.32 days for our original less peaked (i.e. having lower kurtosis) infectivity profile. Serial interval estimates for A/H1N1 2009 influenza range from 1.91 [14], [15] to 2.9 [15], so it is difficult to determine from serial interval data which is more appropriate.

### Related Work

Previous simulation studies have modelled antiviral mitigation strategies, primarily in an H5N1 context [9], [10], [11], [35], [36], [37], [38], [39]. Some of these studies have examined the effect of delays to, and the proportion of cases receiving, antivirals; none have specifically modelled epidemics with characteristics based on data from the A/H1N1 2009 pandemic.

As with our study, that reported in (Ferguson *et al.* 2006) for a similar reproduction number also indicates that both delaying treatment and treating a smaller proportion of cases increases the cumulative attack rate, the maximum daily incidence and the number of antiviral courses required. Additionally, we have quantified this result for in two further antiviral strategies and for a wider range of R_0_ values.

The modelling study reported in [12] examined several logistical constraints on antiviral usage in an influenza pandemic, including the proportion of infected individuals who were treated. Although different modelling assumptions make comparison difficult, a common finding was that for an epidemic with an R_0_ of 1.5, maximum antiviral usage occurs when approximately 50% of cases are diagnosed. Additional results from our study also indicates that this phenomenon is no longer true as R_0_ increases: for R_0_ approximately ≥2.0 increasing diagnosis coverage always leads to increased antiviral usage.

A simulation study that assessed the importance of fast test kits, which would allow diagnosis in one hour as opposed to 12 hours, found that delays in this range were less important than the choice of strategy (e.g. treatment compared to treatment plus prophylaxis), which is consistent with our results [40].

In the study reported in [10] the effect of delaying diagnosis and thus application of antiviral medication an additional day (from 24 to 48 hours) for a combined treatment and extended prophylaxis strategy is also shown to increase both the attack rate and the number of antiviral courses required. This study found that a 24 hour delay assuming a 50% false-positive diagnosis coverage was superior to a 48 hour delay with no false positives. However these results indicate very small resulting attack rates following activation of the antiviral measures, for example reducing the unmitigated cumulative attack rate from 32.6% to between 3.7% and less than 1% for a range of treatment and targeted antiviral strategies, for a reproduction number comparable to that used here. Given the resulting attack rates are perhaps unrealistically small, the effect of delaying antiviral intervention may be more marked than that determined here.

The results presented here are subject to several limitations. We have assumed that the pandemic influenza strain is susceptible to neuraminidase inhibitors and have not attempted to model the effects of antiviral resistance. We have not modelled the potential which antiviral drugs have to reduce or prevent occurrence of serious adverse infection outcomes, which may well make antiviral treatment a worthwhile strategy even in scenarios where only a small stockpile is available or where only small numbers of (serious) cases are diagnosed. Also, our simulated population has demographic, mobility and contact patterns typical of an industralised country setting and may not be applicable to other populations.

### Conclusion

Our evaluation of antiviral drug interventions using a detailed simulation model shows that their effectiveness, and the required antiviral stockpile size, is strongly dependent on (i) the delay occurring between symptom onset and diagnosis, and (ii) the percentage of the infected population being diagnosed, and consequentially benefiting from antiviral administration.

How do the results presented here relate to the actual antiviral interventions implemented during the 2009 A/H1N1 pandemic? Procedures for diagnosing pandemic influenza for the purposes of antivirial treatment or prophylaxis varied considerably between and within countries, and varied according to the stage of the epidemic; however some general conclusions can be drawn.

Evidence suggests that due to the mild nature of the symptoms of the A/H1N1 2009 virus, the proportion of infected people seeking medical attention was low, perhaps on the order of 5%–10% [29], [30]. While antiviral drugs have the potential to reduce or prevent occurrence of serious adverse infection outcomes for treated individuals, our results show that at this low level of diagnosis antivirals have essentially no population-level effect in reducing overall transmission.

Secondly, amongst pandemic plans that called for laboratory testing of suspected cases, a 48-hour turn-around time for testing was anticipated [1], and the experience during 2009 was that average turn-around times were in fact 48-hours or longer (this was the experience, for example, in Western Australia [41]). Our results show that if diagnosis on this timescale is used for the prescription of antivirals for treatment or prophylaxis, their effectiveness is greatly diminished. An alternative diagnosis policy of prescription upon presentation with influenza-like illness (ILI) symptoms would thus improve antiviral effectiveness, but at the risk of higher antiviral usage due to distribution of antivirals for non-influenza ILI.

While the 2009 A/H1N1 pandemic strain has been characterized as mild, our results are equally applicable to a more pathogenic pandemic having similar transmissibility (R_0_ = 1.5). In the case of the emergence of such a virulent strain, it is anticipated that antivirals would be employed on a large scale as part of an effort to contain infection spread.

## Supporting Information

Table S1 Characteristics of simulated baseline epidemics for various R_0_ values.
(0.04 MB DOC)

Table S2 Effectiveness of antiviral intervention strategies assuming seasonal influenza age-specific attack rates and R_0_ = 1.5.
(0.04 MB DOC)

Figure S1 Effects of diagnosis delay for various R_0_ values.
(0.07 MB PDF)

Figure S2 Effects of diagnosis ratio for various R_0_ values.
(0.12 MB PDF)

Text S1 Details of additional sensitivity analyses.
(0.45 MB PDF)
