# Supplementary material for: The Impact of Case Diagnosis Coverage and Diagnosis Delays on the Effectiveness of Antiviral Strategies in Mitigating Pandemic Influenza A/H1N1 2009
Source: PLoS One. 2010 Nov 3;5(11):e13797. doi: 10.1371/journal.pone.0013797 (PMC2972206; doi:10.1371/journal.pone.0013797)
Supplement: Table S1 — Characteristics of simulated baseline epidemics for various R0 values. (0.04 MB DOC) [file pone.0013797.s001.doc]

The Impact of Case Diagnosis Ratio and Diagnosis Delays on the Effectiveness of Antiviral Strategies in Mitigating Pandemic Influenza A/H1N1 2009

**Supporting Information Table S1**

**Characteristics of simulated baseline epidemics for various R_0_** values

|  | **R_0_** | | | | | | | |
| --- | --- | --- | --- | --- | --- | --- | --- | --- |
|  | 1.2 | | 1.5 | | 2.0 | | 2.5 | |
| **characteristic** | **Mean** | **S.D.** | **Mean** | **S.D.** | **Mean** | **S.D.** | **Mean** | **S.D.** |
| Final infection rate (%) | 16.5 | 1.17 | 32.0 | 0.87 | 48.0 | 0.51 | 58.4 | 0.36 |
| Final symptomatic attack rate (%) | 12.8 | 0.90 | 24.5 | 0.65 | 36.4 | 0.39 | 43.8 | 0.29 |
| Peak daily symptomatic incidence (per 10,000) | 33 | 5 | 109 | 9 | 274 | 12 | 450 | 17 |
| Peak symptomatic population (%) | 1.7 | 0.27 | 5.8 | 0.43 | 14.3 | 0.49 | 22.3 | 0.49 |
| Peak incidence day | 51 | 11.1 | 35 | 4.0 | 25 | 2.0 | 19 | 1.9 |
| Serial interval (days) | 2.36 | 1.60 | 2.32 | 1.65 | 2.21 | 1.66 | 2.11 | 1.70 |

The fundamental transmission probability beta was adjusted to give epidemics with measured R_0_ values of 1.2, 1.5, 2.0 and 2.5. Means and standard deviations (S.D.) for each epidemic statistic (except for serial interval, see below) were calculated from 40 independent simulation runs with all stochastic choices take from a different random number stream. The standard deviation of the serial interval is for the distribution of individual serial intervals form all infections accross the 40 simulation runs (not the standard deviation of the mean serial intervals from the 40 simulation runs).
