# Supplementary material for: The Impact of Case Diagnosis Coverage and Diagnosis Delays on the Effectiveness of Antiviral Strategies in Mitigating Pandemic Influenza A/H1N1 2009
Source: PLoS One. 2010 Nov 3;5(11):e13797. doi: 10.1371/journal.pone.0013797 (PMC2972206; doi:10.1371/journal.pone.0013797)
Supplement: Table S2 — Effectiveness of antiviral intervention strategies assuming seasonal influenza age-specific attack rates and R0 = 1.5. (0.04 MB DOC) [file pone.0013797.s002.doc]

The Impact of Case Diagnosis Ratio and Diagnosis Delays on the Effectiveness of Antiviral Strategies in Mitigating Pandemic Influenza A/H1N1 2009

**Supporting Information Table S2**

**Effectiveness of antiviral intervention strategies assuming seasonal influenza age-specific attack rates and R_0_** = 1.5

|  | **final symptomatic attack rate (%)** | | | | | | **peak daily incidence (per 10,000)** | | | | | |
| --- | --- | --- | --- | --- | --- | --- | --- | --- | --- | --- | --- | --- |
| baseline | 32.5 | | | | | | 121 | | | | | |
| **intervention strategy** | **diagnosis delay** | | | **diagnosis ratio** | | | **diagnosis delay** | | | **diagnosis ratio** | | |
|  | 0 h | 24 h | 48 h | 10% | 50% | 90% | 0 h | 24 h | 48 h | 10% | 50% | 90% |
| T | 20.8 | 26.5 | 28.6 | 31.2 | 26.5 | 21.4 | 57 | 87 | 97 | 112 | 87 | 61 |
| T+SC | 17.2 | 23.1 | 24.8 | 26.6 | 23.1 | 17.8 | 33 | 53 | 58 | 61 | 53 | 38 |
| T+H | 11.9 | 18.9 | 22.3 | 29.4 | 18.9 | 9.8 | 21 | 46 | 62 | 99 | 46 | 18 |
| T+H+SC | 9.2 | 15.4 | 19.3 | 25.2 | 15.4 | 7.7 | 14 | 27 | 38 | 55 | 27 | 12 |
| T+H+E | 7.2 | 13.1 | 17.2 | 26.7 | 13.1 | 5.5 | 12 | 25 | 37 | 83 | 25 | 9 |
| T+H+E+SC | 6 | 10.9 | 15.1 | 23.4 | 10.9 | 4.7 | 9 | 15 | 25 | 48 | 15 | 7 |

Final symptomatic attack rate (as % of population) and peak daily symptomatic incidence (per 10,000) are given for different intervention strategies, diagnosis delays and diagnosis ratios. Intervention strategies are abbreviated as follows: T – antiviral treatment of diagnosed cases, H – prophylaxis of household of diagnosed cases, E – prophylaxis of school or work contacts of diagnosed cases, SC – four weeks of school closure. Where diagnosis delay differs from 24 hours, diagnosis ratio is 50%; where diagnosis ratio differs from 50%, diagnosis delay is 24 hours.
