# Supplementary material for: The Impact of Case Diagnosis Coverage and Diagnosis Delays on the Effectiveness of Antiviral Strategies in Mitigating Pandemic Influenza A/H1N1 2009
Source: PLoS One. 2010 Nov 3;5(11):e13797. doi: 10.1371/journal.pone.0013797 (PMC2972206; doi:10.1371/journal.pone.0013797)
Supplement: Figure S2 — Effects of diagnosis ratio for various R0 values. (0.12 MB PDF) [file pone.0013797.s004.pdf]

The Impact of Case Diagnosis Ratio and Diagnosis Delays on the Effectiveness of Antiviral Strategies in Mitigating Pandemic Influenza A/H1N1 2009

Support Information Figure S2

Final attack rate (%) | Peak daily incidence (per 10,000) | Antiviral usage (% of population size)

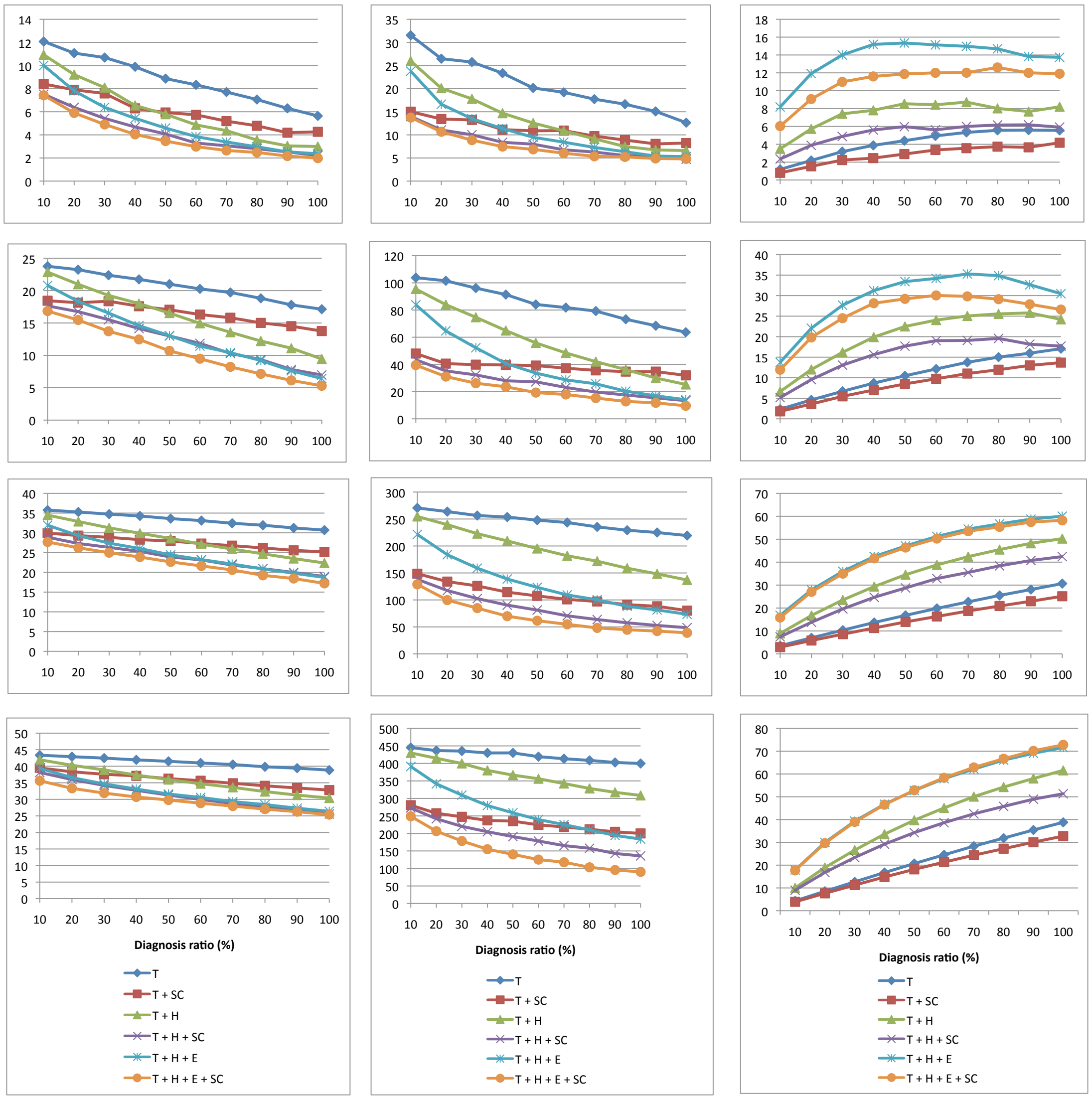


R0 = 1.2

R0 = 1.5

R0 = 2.0

R0 = 2.5
