# Supplementary material for: The Impact of Case Diagnosis Coverage and Diagnosis Delays on the Effectiveness of Antiviral Strategies in Mitigating Pandemic Influenza A/H1N1 2009
Source: PLoS One. 2010 Nov 3;5(11):e13797. doi: 10.1371/journal.pone.0013797 (PMC2972206; doi:10.1371/journal.pone.0013797)
Supplement: Text S1 — Details of additional sensitivity analyses. (0.45 MB PDF) [file pone.0013797.s005.pdf]

The Impact of Case Diagnosis Coverage and Diagnosis Delays on the Effectiveness of Antiviral Strategies in Mitigating Pandemic Influenza A/H1N1 2009

Supporting Information Text S1 : Sensitivity Analyses

Part 1 : Constant AVE_i vs Declining AVE_i

Our baseline assumption was that once an individual was receiving antiviral treatment, that individual's infectivity would be reduced by the antiviral effectiveness (AVE_i) value of 66% [1]. It may be the case however that antiviral effectiveness may be dramatically reduced if treatment is delayed. To model this possibility, we conducted an alternative set of diagnosis-delay experiments where we assumed that AVE_i would decrease by 50% for each 24-hour delay after symptoms developed. Since we simulated delays in increments of 12 hours, this gave AVE_i values of 66%, 47%, 33%, 23% and 16.5% for delays of 0, 12, 24, 36 and 48 hours respectively. All other simulation parameters were as reported in the main text.

Figure S1.1 illustrates the infectivity profiles for delays of 0, 24 and 48 hours for both the constant AVE_i and declining AVE_i assumptions.

Figure S1.2 shows the final attack rates for diagnosis delays from 0 to 48 hours. As noted in the main text, the declining AVE_i assumption does not lead to significant additional loss of antiviral effectiveness.

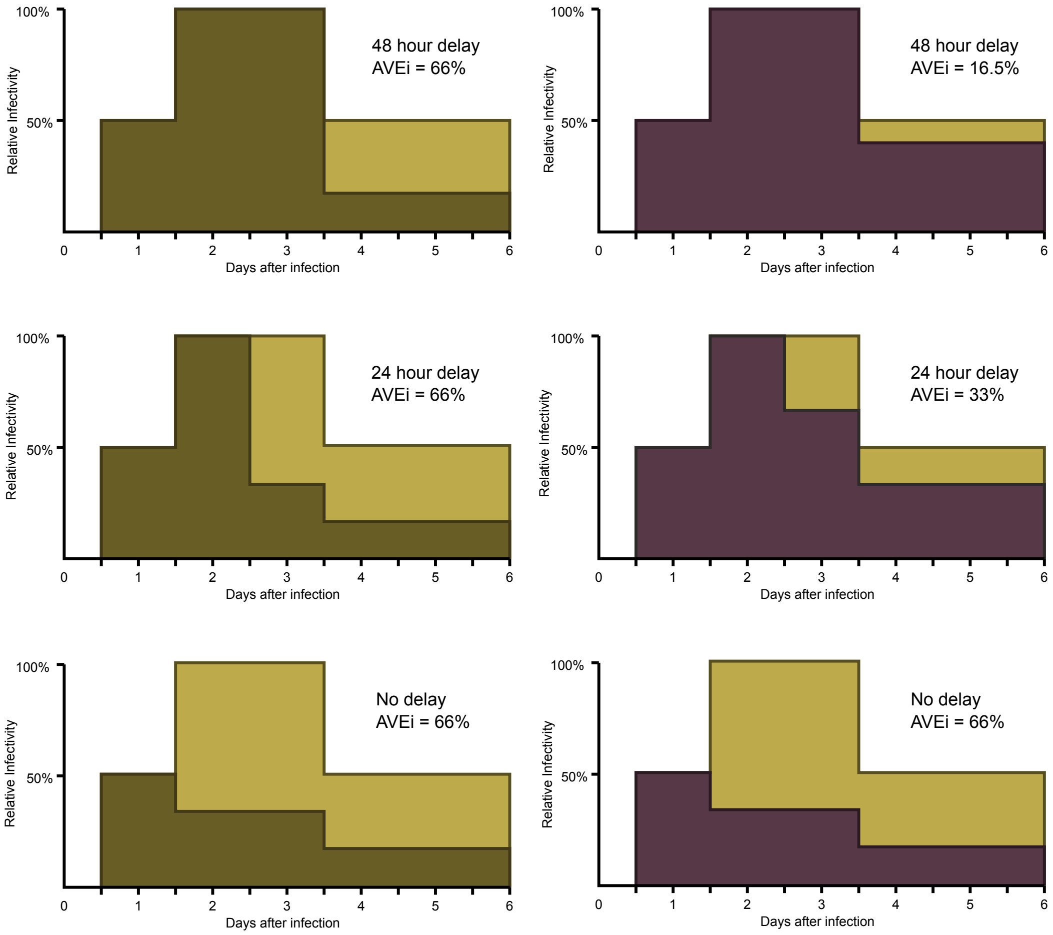

Figure S1.1 The light brown areas represent the baseline infectivity profile assuming no antiviral treatment, the dark brown areas represent the infectivity profile assuming constant AVE_i, while the purple area represent the infectivity profile assuming declining AVE_i.

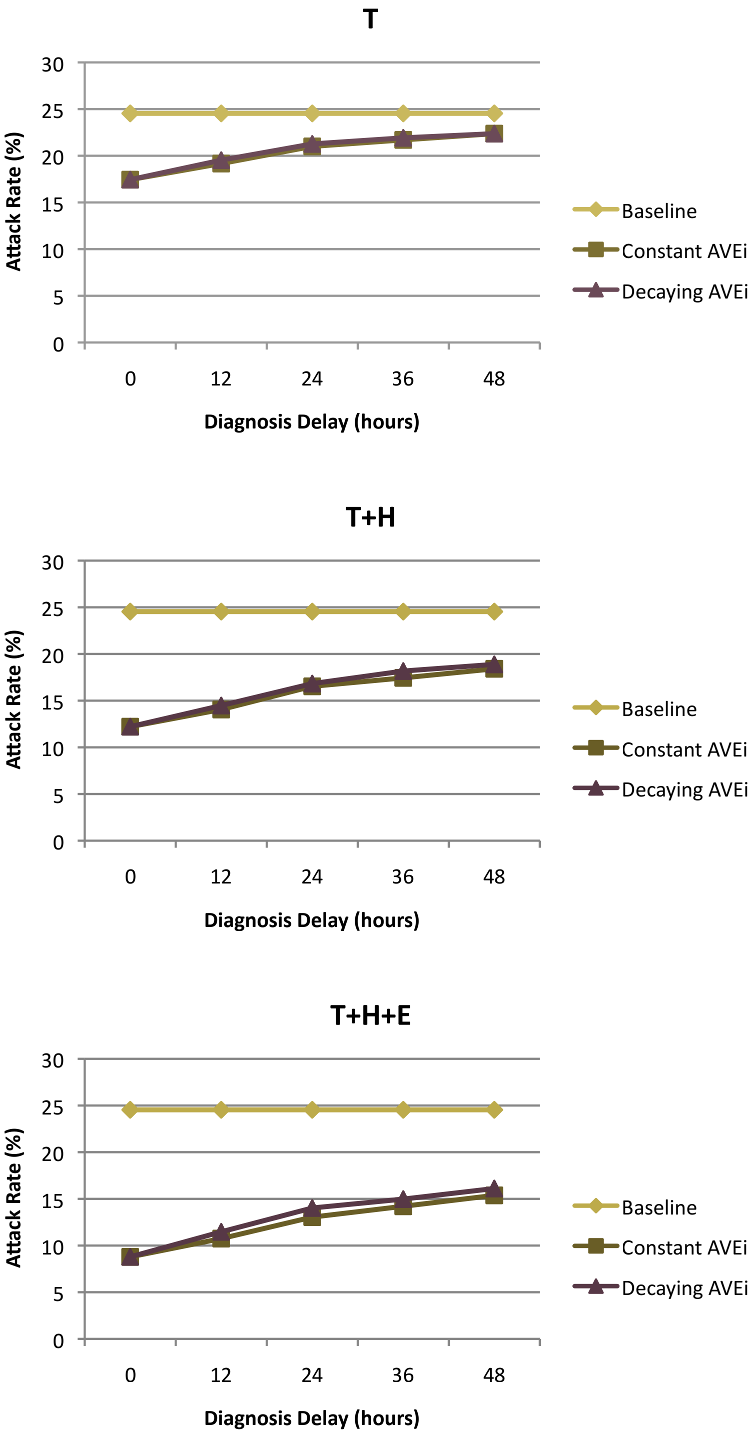

Figure S1.2 Shown are final attack rates for the treatment-only (T) strategy (top), treatment plus household prophylaxis (T+H) strategy (middle) and treatment plus household plus extended prophylaxis strategy (bottom). Colours are the same as for Figure S1.1: light brown is no antivirals, dark brown is the constant AVE_i assumption, purple is the declining AVE_i assumption.

Part 2 : Alternative Infectivity-Over-Time Profiles

In order to determine the sensitivity to our diagnosis delay results to the shape of the individual infectivity profile (how an individual's infectiveness varies over time after infection), we repeated our diagnosis delay experiments with 4 additional alternative infectivity profiles. These are pictured in Figure S1.3 and are as follows:

1. The baseline assumption: asymptomatic and post-symptomatic infectivity 0.5, infectivity beginning after 12 hours, peak infectivity from 36 hours to 84 hours, recovery after 144 hours.
2. Long infection: asymptomatic and post-symptomatic infectivity 0.5, infectivity beginning after 24 hours, peak infectivity from 48 to 96 hours, recovery after 168 hours.
3. Short infection: asymptomatic and post-symptomatic infectivity 0.5, infectivity beginning after 12 hours, peak infectivity from 24 to 52 hours, recover after 144 hours.
4. High peak infectiviy: asymptomatic and post-symptomatic infectivity 0.25, timing as for the baseline (1 above).
5. Short with high peak infectivity: asymptomatic and post-symptomatic infectivity 0.25, timing as for "short" (3 above).

For each profile the basic infectivity probability β was determined such that the unmitigated epidemic has a R_0 value of 1.5, to match the main experiment series. The characteristics of the resulting unmitigated epidemics are given in table below.

Table : Alternative infectivity profile epidemic characteristics

Infectivity Profile	Serial Interval (days)	Peak incidence day	peak daily incidence (per 10,000)	Final attack rate (%)
Baseline	2.32	35	109	24.6
Peaked	2.27	38	98	23.5
Short	1.99	37	89	21.3
Long	2.94	47	83	23.7
Short-Peaked	1.85	36	90	21.3

Figure S1.4 Shows the final attack rates as a function of diagnosis delay for each infectivity profile and for the T, T+H, and T+H+E intervention strategies.

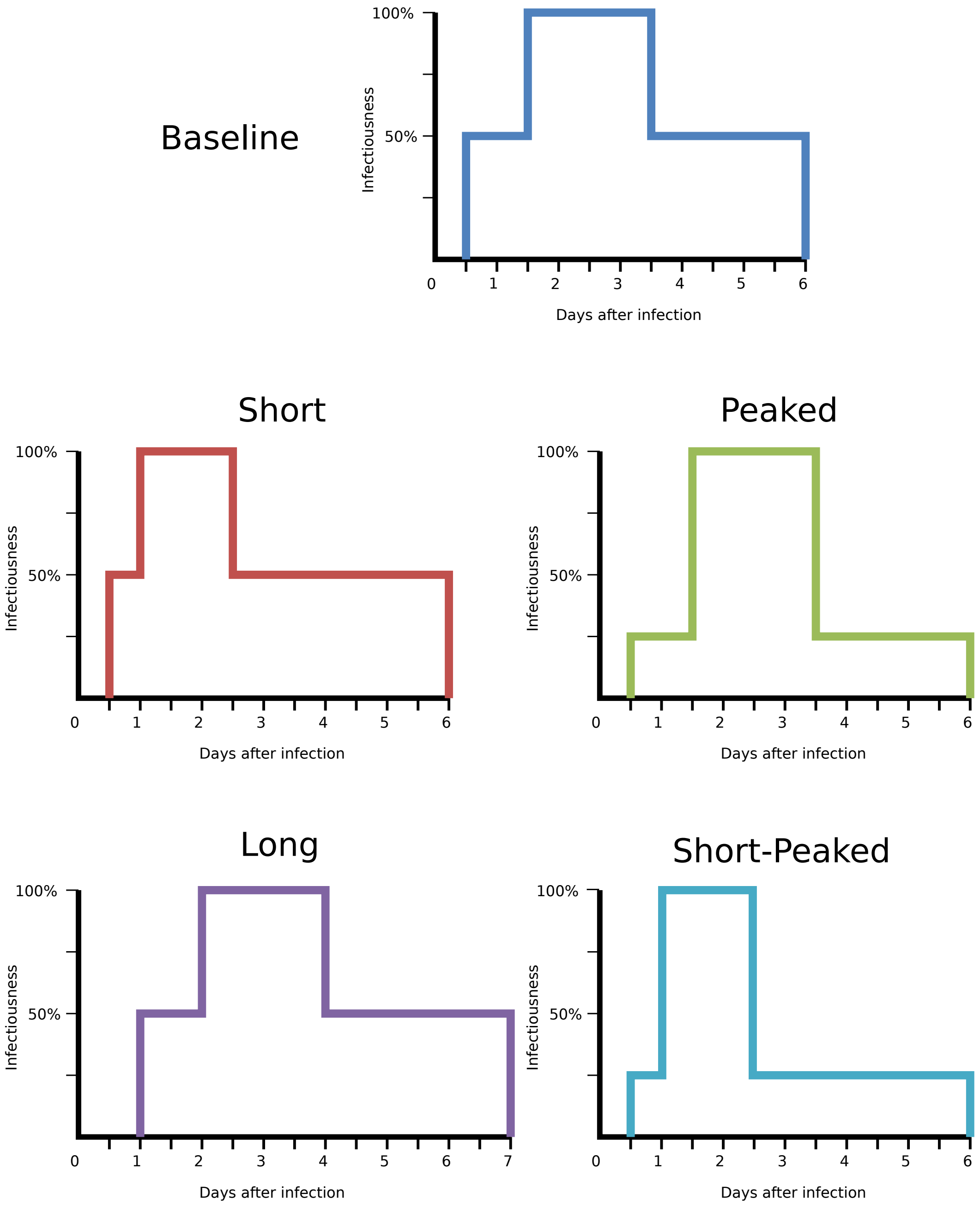

Figure S1.3 Alternative infectivity profiles.

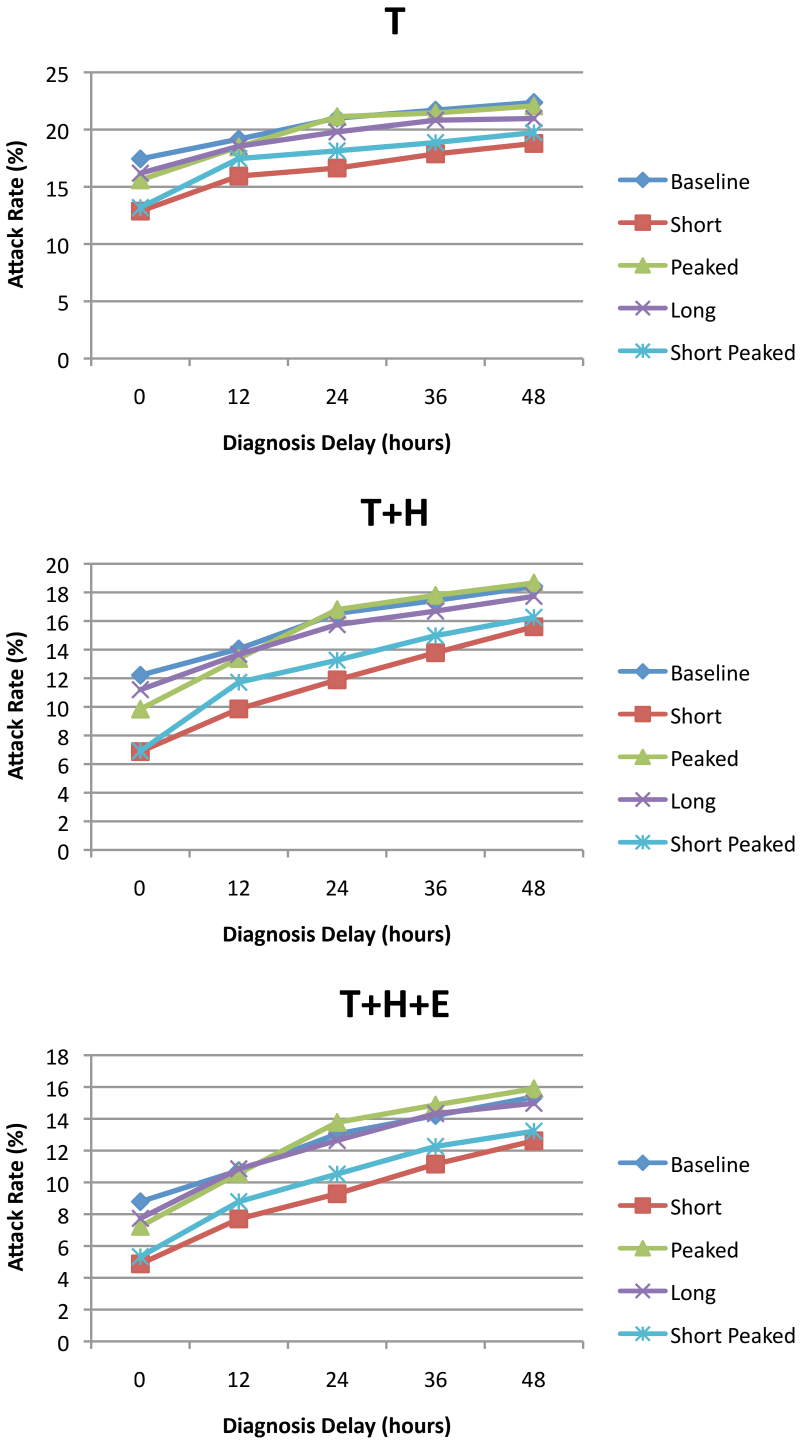

Figure S1.4 Final attack rates as a function of diagnosis delay for each infectivity profile and for the T (top), T+H (middle), and T+H+E (bottom) intervention strategies. Infectivity profiles are coloured as for Figure S1.3 .

References

1. Yang Y, Longini IM, Jr., Halloran ME (2006) Design and evaluation of prophylactic interventions using infectious disease incidence data from close contact groups. Appl Statist 55: 317-330.
